# Force Measurements of TCR/pMHC Recognition at T Cell Surface

**DOI:** 10.1371/journal.pone.0022344

**Published:** 2011-07-22

**Authors:** Pierre-Henri Puech, Damien Nevoltris, Philippe Robert, Laurent Limozin, Claude Boyer, Pierre Bongrand

**Affiliations:** 1 Laboratoire Adhésion et Inflammation (LAI), Parc Scientifique et Technologique de Luminy, Marseille, France; 2 Inserm UMR 600, Parc Scientifique et Technologique de Luminy, Marseille, France; 3 CNRS UMR 6212, Parc Scientifique et Technologique de Luminy, Marseille, France; 4 Université de la Méditerranée, Parc Scientifique et Technologique de Luminy, Marseille, France; 5 Hôpital de la Conception, Service d'Immunologie, Marseille, France; 6 Centre d'Immunologie de Marseille Luminy (CIML), Université de la Méditerranée, UMR 6546, Parc Scientifique et Technologique de Luminy, Marseille, France; 7 Inserm UMR 631, Parc Scientifique et Technologique de Luminy, Marseille, France; 8 CNRS UMR 6102, Parc Scientifique et Technologique de Luminy, Marseille, France; Swiss Federal Institute of Technology Zurich, Switzerland

## Abstract

The rupture forces and adhesion frequencies of single recognition complexes between an affinity selected peptide/MHC complex and a TCR at a murine hybridoma surface were measured using Atomic Force Microscopy. When the CD8 coreceptor is absent, the adhesion frequency depends on the nature of the peptide but the rupture force does not. When CD8 is present, no effect of the nature of the peptide is observed. CD8 is proposed to act as a time and distance lock, enabling the shorter TCR molecule to bridge the pMHC and have time to finely read the peptide. Ultimately, such experiments could help the dissection of the sequential steps by which the TCR reads the peptide/MHC complex in order to control T cell activation.

## Introduction

A key step of the immune response is the detection by T lymphocytes, thanks to their T cell receptor (TCR), of foreign peptides bound to major histocompatibility complex molecules (pMHC) on the surface of antigen presenting cells (APCs). In addition to its prominent physiological importance, the interaction of the TCR with pMHC raises enormous interest due to a number of extraordinary features. (i) Recognition is exquisitively specific since T lymphocytes have been reported to detect a single cognate pMHC complex on APCs exposing many tens of millions of proteins on their membranes [1], [2]. (ii) The TCR repertoire must be rich enough to cope with many millions of potentially harmful structures and specific enough to avoid autoimmune phenomena. (iii) Recognition and subsequent activation must be rapid enough to occur during a typical contact ranging in duration from seconds to minutes between an APC and a T lymphocyte [3]. (iv) Recognition is not an all-or-none event since it may generate widely different outcomes, ranging from full lymphocyte activation to anergy following minute variations of the peptide antigen sequence [4].

The binding of a cognate pMHC by the TCR is thought to involve the participation of a co-receptor that may be CD4 or CD8 for class II and class I MHC respectively. While a well established role of CD4 or CD8 is to enhance signaling cascades [5], [6], these molecules also influence binding by acting as low affinity receptors [7], having a high association rate *per se*, increasing the association rate of soluble pMHC to T lymphocytes and decreasing the dissociation rate [8]. The interaction between CD8 and TCR is complex, since TCR engagement may activate CD8-mediated adhesion [9] and CD8 may modulate TCR avidity [10].

Signal generation as a consequence of pMHC/TCR interaction is difficult to explain on the basis of a conformational change [11], [12]. Rather, interaction outcome was reported to depend on the lifetime of individual TCR / MHC complexes [13]–[15]. Some experiments supported the intriguing hypothesis that signaling might also involve force generation at the lymphocyte / APC interface [16], [17].

In order to gain new insight into the mechanisms of TCR-mediated lymphocyte activation, it is essential to relate the outcome of the lymphocyte/APC interaction to the physical properties of TCR/pMHC interactions such as the kinetic parameters or the forces of the TCR / pMHC interaction. Much work has been done to measure these interactions using recombinant elements in soluble phase with surface plasmon resonance [13], [18]. However, it is well recognized that molecular interactions between surface-bound, especially cell membrane bound, receptors are influenced by several parameters, e.g. force sensitivity, molecular flexibility or steric effects, that are not accounted for by measurements made in solution [19] and that may be profoundly influenced by active cellular processes [20]. Therefore, the use of powerful biophysical tools such as atomic force microscopy applied to molecular studies is warranted to provide an accurate characterization of molecular interactions between TCRs and pMHCs on the cell surface.

An Atomic Force Microscope (AFM) in force mode uses cantilever deflection to measure the forces that are exerted on the lever extremity. The sensitivity of force determination is limited by the thermal noise of the system [21]. AFM has proved to be a very well suited technique for measuring molecular interactions, from single molecule unbinding events [22], [23] to single cell detachment [24], [25].

In this article, we assessed the respective roles of the TCR and CD8 co-receptor during the first hundred milliseconds following the contact of a model surface decorated with pMHC and a living T cell. We used two hybridoma lines expressing similar levels of BM3.3 TCR with (line C3.CD8) or without (line 4C8.98) the CD8 coreceptor. BM3.3 TCR recognizes the pBM1 peptide bound to allogenic MHC Class I [26] and is powerful enough (EC_50_∼10^−14^ – 10^−11^ M, K_d_∼2.6 10^−6^ M as measured by SPR), such that it can under certain conditions induce CD8 independent T-cell activation [27], [28]. Thus, comparing the interaction between this TCR and its cognate ligand (pBM1 peptide presented by H-2K^b^) or a non-activating peptide (OVA presented by H-2K^b^) has relevance since currently available methods of studying single bond rupture may not be sensitive enough to analyze the interaction of "weak" TCRs with their ligands. Finally, the use of an hybridoma such as C3.CD8 expressing high levels of CD8 as compared to TCR molecules should be optimally suited to detect an additional effect of CD8 as compared to CD8-independent responses.

This article is the first report to the authors's knowledge, of a direct monitoring of TCR/pMHC interaction at the single bond level and in terms of forces on the surface of living cells.

## Results

**Figure 1:**
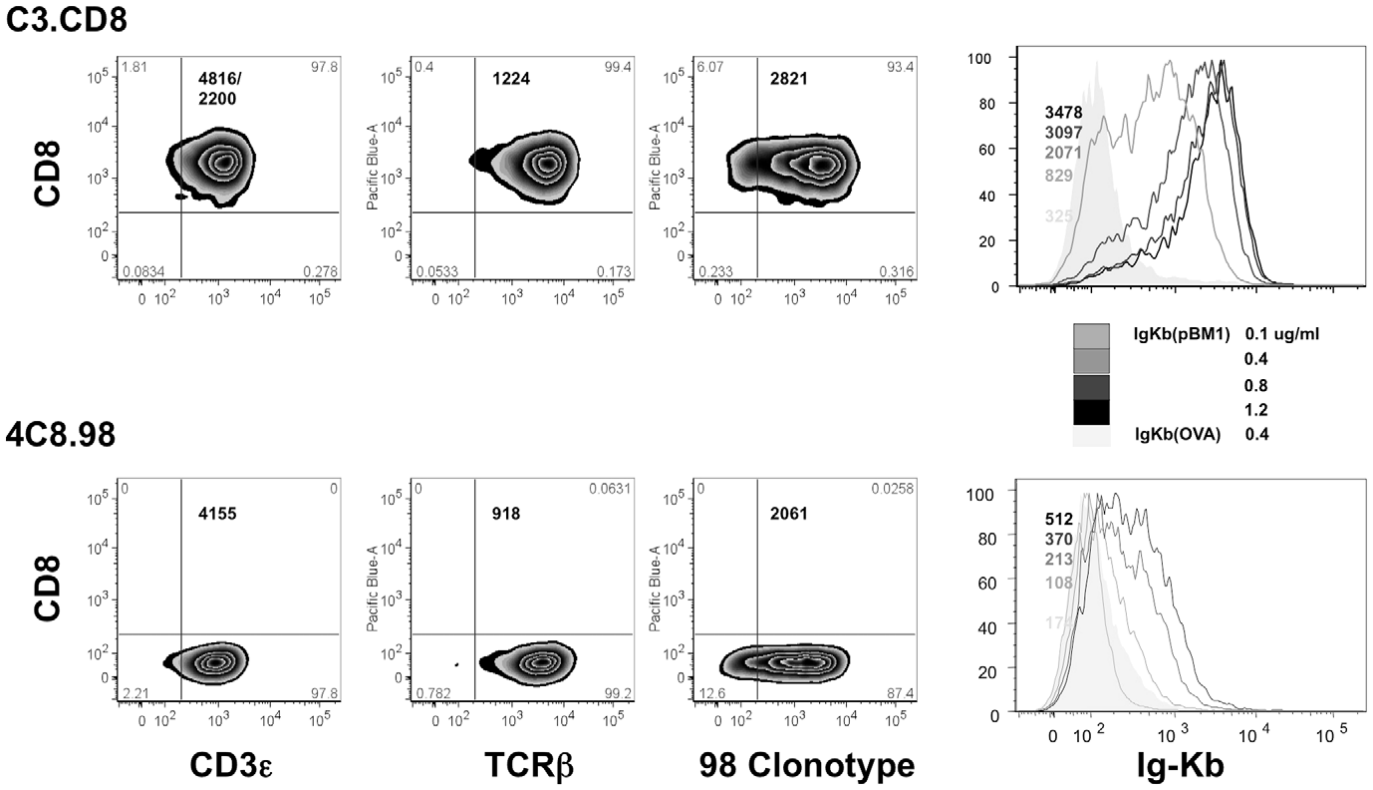
Flow cytometry experiments. On the first six panels (left to right), numbers represent the mean fluorescence for TCR or CD3/ CD8 using relevant antibodies (see Material and methods). On the last two panels (right), dilutions of the H-2Kb DimerX loaded with OVA or pBM1 peptide are expressed in µg/mL.

Using flow cytometry, we verified that both cell lines employed in this study expressed the desired molecules on their surface (TCR, CD3 and CD8) and that TCR levels were similar (Fig. 1, first six panels). Using the recombinant fusion protein Dimer X to expose peptide loaded H-2K^b^, we showed that TCR binding was peptide specific (low binding for OVA, stronger binding for pBM1) and dose dependent. The presence of CD8 at the cell surface greatly enhanced the binding of the pMHCs to the cell surface (Fig. 1, last two panels). We measured by cytometry that 75% of C3.CD8 cells express both CD8α and CD8β on their surface (Fig. S1). CD8 on C3.CD8 cell line is composed of both CD8αβ and CD8αα dimers. In view of previous reports [29], CD8α may be considered as responsible for the interaction between CD8 dimers and MHC molecules.

**Figure 2:**
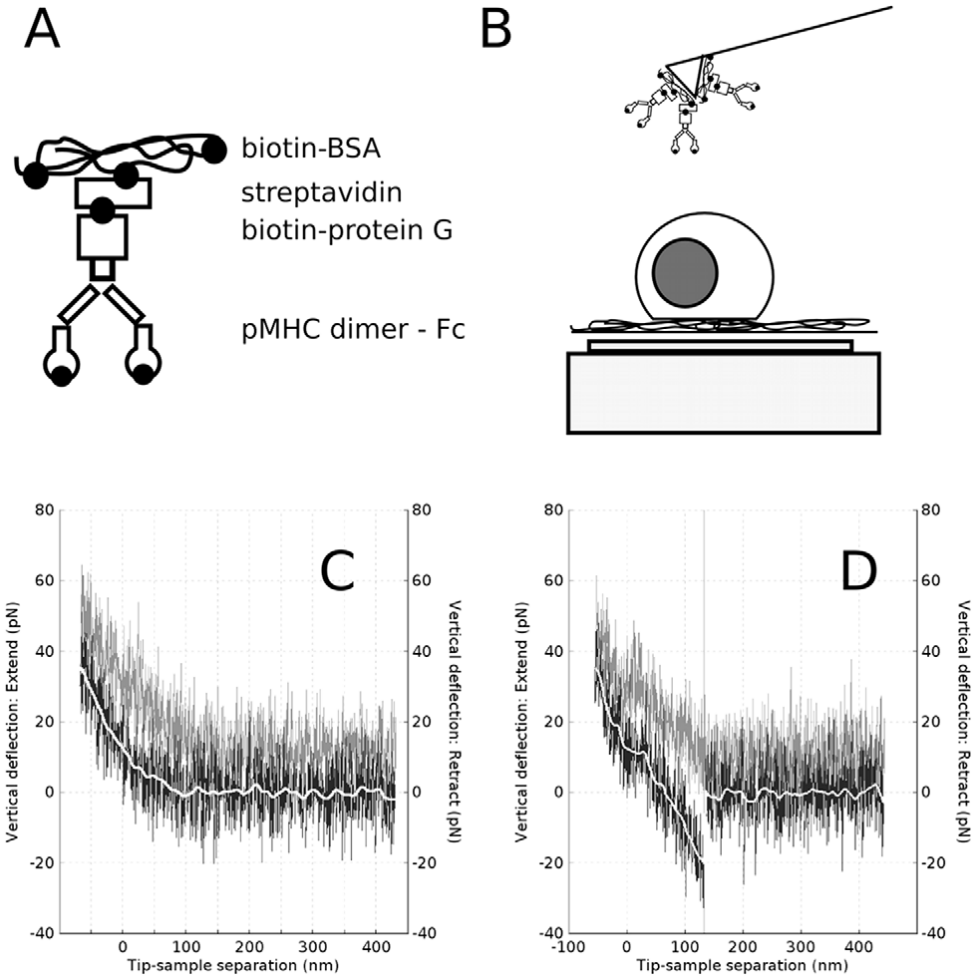
AFM force mode experiments. A : Schematic representation of the cantilever decorating structure employed to favor a correct presentation of the pMHC. B : Schematic of the recognition force measurements on polylysine adhered T hybridomas using a pMHC decorated AFM lever (not to scale). C : Typical force curve (*F* vs. piezo position – pushing, black and pulling, grey) for a contact force of 50 pN, a contact time of 0 sec and at a speed of *v_press_ = v_pull_* = 1 µm/sec showing *no* adhesion. D : Typical force curve, taken in the same conditions as C, showing a single adhesion event. The white line is a 45 points running average of the noisy force curve used to automatically detect and measure the force jump using JPK-IP software (vertical grey line).

Cantilever tips decorated with pMHC were used to investigate the molecular recognition at the cell surface. By bringing a tip and a cell into contact and separating them with controlled speed and force, the force exerted at the tip extremity can be assessed by measuring the bending of the cantilever (Fig. 2A,B and Fig. S2). To ensure that the cantilever tip contacted the cell membrane and not only the cell glycocalyx, we performed micro-mechanical measurements on the hybridoma (Fig. S3). We observed that the low contact force used here (50 pN) was sufficient to bring the molecules on the tip (pMHCs) in close contact with their partners on the cell membrane (TCR / CD8) since (i) the Young modulus of the cells was measured to be the same as for a tenfold higher contact force and (ii) reproductible contact between the tip and the cell was obtained for such a small contact force. A minimal contact time was chosen to have low adhesion frequencies and thus be able to investigate mainly single molecules interactions following statistical arguments : when adhesion frequency is lower of 30% it may be assumed that 80% of the binding events represent single molecule events [30]. Typical force curves, without or with an interaction, are shown on Fig. 2C and D respectively.

A short contact time is highly relevant to dissecting the first steps of the molecular recognition between pMHC, TCR and/or CD8 (see Discussion). This contact time, *t_c_*, can be estimated, in average, as *t_c_ = t_mech_ + t_AFM_* , where *t_AFM_* is the AFM macroscopic experimental time, here set to 0 sec. *t_mech_* is the effective contact time imposed by the mechanical properties of the cells described by the Young modulus, *E*, the chosen contact force, *F_c_*, and cantilever speed, *v*. It can be calculated as *t_c_ = t_mech_ = *2 *d / v* where *d* is the cell indentation. Using the Hertz model for a pyramidal tip of half angle *α* = 35° [31], we have *d* ^2^ *= (*4*F_c_ (1-υ* ^2^ *)) / (*3*E* tan *α).* Assuming incompressibility (Poisson ratio *υ = *.5) and taking *F_c_* = 50 pN, *E*∼3000 Pa and *v* = 1 µm/sec, one obtain a contact time t_c_∼300 msec.

The first striking observation was that the unbinding forces were low as compared to the forces measured with classical adhesion molecules, such as integrins and cadherins [21], [24], [32]–[34]. The detachment forces measured here were of the same magnitude as the apparent noise of the force curves (measured to be on the order of 10 pN). This implied that averaging is necessary to precisely detect the force jumps (Fig. 2C, D, white line, see Methods).

**Figure 3:**
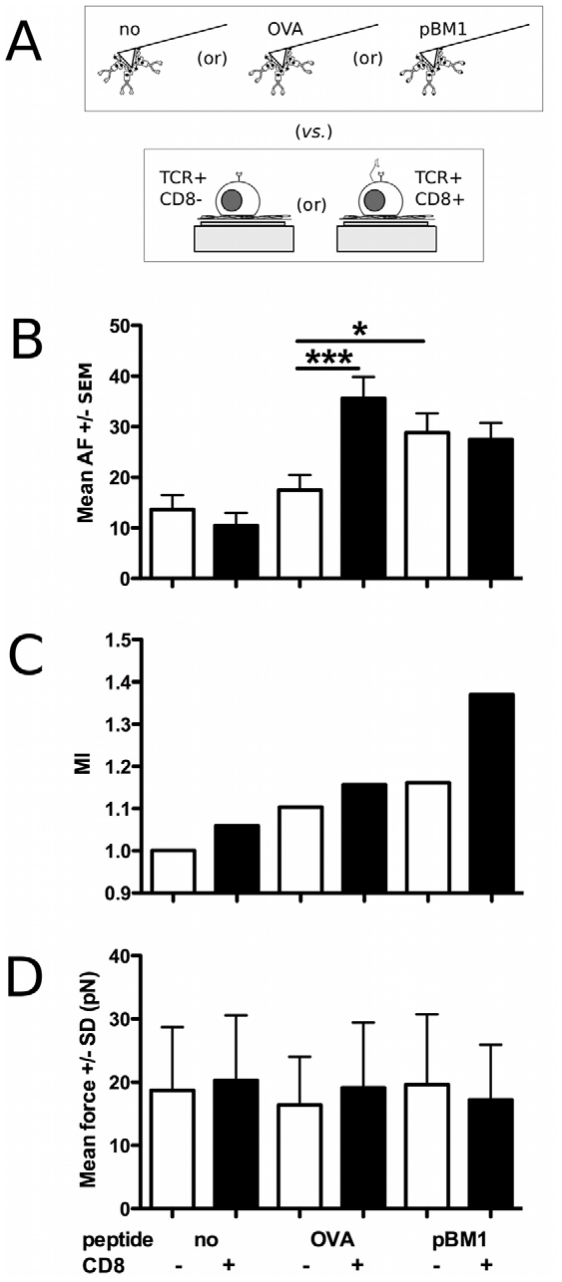
AFM force mode experiments. A. Schematics of the experiments leading to the data presented in B–D. B : Adhesion frequency, *AF* (+/− SEM), per cell and C : index of multiplicity, *MI,* vs. peptide, as a function of cell type for an apparent contact time of 0 sec and a contact force of 50 pN. Stars depict significantly different values (t-test, *p*<0.05). D : Average rupture force of single complex ruptures, extracted from the histograms (+/− SD), as a function of cell type and peptide. The values are *not* significantly different (ANOVA + post-test, *p*>0.05). For the number of cells and force curves that have been analysed, see Table 1.

Six combinations of interacting surfaces were studied with the same pulling speed (1 µm/sec ; Table 1 and Fig. 3A). CD8− and CD8+ cells were contacted with cantilevers presenting one of two non activating, empty H-2K^b^ or OVA:H-2K^b^, or one activating system, pBM1:H-2K^b^. In addition to the force of single de-adhesion jumps, we recorded the total number of force curves obtained, *N_curves_*, the number of force curves with at least one force jump, *N_adhesion_*, and the sum of the number of force jumps per force curve, *N_jumps_*.

**Table 1:** Adhesion/recognition data: number of curves (number of cells ; days) as a function of cell type and peptide.

Cells / peptide	empty	OVA	pBM1
4C8.98 (TCR+ / CD8−)	175 (20 ; 2)	269 (34 ; 2)	195 (24 ; 2)
C3.CD8 (TCR+ / CD8+)	154 (17 ; 2)	245 (33 ; 2)	525 (62 ; 4)

To assess the specificity of the measured interaction with regards to the cytometry data, the adhesion frequency for each cell, *AF = N_adhesion_/N_curves_*, and the corresponding averages over the different cells of the same type were obtained (Fig. 3B). A multiplicity index of the unbinding events, namely *MI = N_jumps_/N_adhesion_* was calculated for each condition (all cells pooled, Fig. 3C). The presence or absence of a large amount of peptide in the solution while performing the force measurements did not significantly affect the results (not shown), excluding the possibility of a significant loss of the peptide for the time scale of the experiments and cantilever storage.

For the two cell lines, *AF* was measured to be the lower when the MHCs were presenting no peptide (13.7+/− 2.8 % and 10.5+/− 2.5 % for CD8− and CD8+ cells respectively) and was found of similar value (17.5+/− 3.0 %) for OVA:H-2K^b^ presented to CD8− cells (Fig. 3B). *AF* was observed to be significantly higher for OVA:H-2K^b^ presented to CD8+ cells (35.6+/− 4.3%). Consistent with the known capacity of pBM1:H-2K^b^ to activate BM3.3 T lymphocytes in absence of CD8 [27], CD8- cells displayed higher *AF* for pBM1:H-2K^b^ (28.8+/− 3.8 %) than for OVA:H-2K^b^ . Interestingly, *AF* was found statistically similar for OVA:H-2K^b^ and pBM1:H-2K^b^ , (27.5+/− 3.3%) presented to CD8+ cells and of the same magnitude as for pBM1:H-2K^b^ presented to CD8- cells.

As a control, we performed experiments where BW cells, which lack TCR and CD8 (see Methods), were used. They lead to low *AF,* similar to those measured for the empty H-2K^b^ situation described above : 17.1+/− 4.8 % for empty H-2K^b^ , 10.6+/− 1.9 % for OVA:H-2K^b^ and 14.4+/− 1.9 % for pBM1:H-2K^b^. Cantilevers bearing no H-2K^b^ presented to CD8+ cells lead to similar *AF* (13.2 % for biotin-BSA, 13.5 % for streptavidin and 6 % for protein-G decorated levers). This allowed us to conclude that, for an AFM contact time of 0 sec and a contact force of 50 pN, a residual *AF* of 10–15% originated from non specific interactions.

In summary, *AF* were peptide dependent in the absence of CD8. In presence of CD8, the presentation of a peptide by the MHC was required to obtain high *AF*, but these *AF* did not discreminate between peptide antigens. When increasing the contact time from 0 sec to 100 msec and 1 sec, *AF* increases as expected for CD8- and CD8+ cells for the three peptides (Fig. S5). The ranking is similar as in the case of 0 sec contact (pBM1 > OVA > no peptide for a given cell type, and CD8+ ≥ CD8- for a given peptide). The increase in *AF* is monotoneous for CD8+ cells but not for CD8-.

*MI* was observed to be 1.00 and 1.06 when the empty H-2K^b^ was presented to CD8- and CD8+ cells respectively (Fig. 3C). For OVA:H-2K^b^, *MI* stayed closer to 1 for CD8- cells (1.10) in regard to CD8+ ones (1.16). *MI* increased when pBM1:H-2K^b^ was used in comparison to OVA:H-2K^b^, slightly for CD8- cells (1.16) and strongly for CD8+ ones (1.37). This indicated that, on average, the number of detectable detachment events per force curve increased when a peptide was present, and this number was higher with an activating peptide.

Surprisingly, the extracted average force values for single force jumps were *not* significantly dependent on either the cell line or the presented peptide, when present (Fig. 3D). The forces were similar to the ones measured using BW cells (not shown). Double jumps occuring in the same force curve for pBM1:H-2K^b^ presented to CD8+ cells exhibited similar magnitude as the single jumps (Fig. S4), independently of their separation in distance. Aside, forces of single jumps are not varying when the contact time is increased up to 1sec (Fig. S5). Force and its variation relative to the presented peptide, as quantified here ie. at single molecule scale, has never been reported in literature for the T cell recognition machinery.

## Discussion

In this report, atomic force microscopy (AFM) in force mode [21] was used to measure the unbinding forces of single TCR / pMHC molecules on living murine T hybridoma cell surfaces. MHCs bound to two peptides of known activity and “empty” MHCs were used to probe T cells expressing a TCR with or without its CD8 coreceptor. AFM allowed us to study interactions in a time short enough to minimize active cell phenomena that have been recently reported to profoundly influence TCR/pMHC interaction at the cell surface [20], [35].

Flow cytometry experiments verified that the cell lines had similar TCR levels, together with the desired expression of CD8, and that the binding, at equilibrium, of pMHCs was peptide and dose dependent. The AFM experiments revealed that the frequency of adhesion events, *AF*, linked to the on rate, *k_on_*, of the recognition reaction, [23], [36], but not the rupture forces, related to its off rate, *k_off_*, [37], [38] was dependent on the nature of the presented peptide. The case of CD8- cells allowed us to conclude that we observed specific and peptide dependent events. The low adhesion frequencies supported the hypothesis that these events were mainly due to single molecule recognition [30].

In line with previous reports and due to the very low forces measured, the rupture events recorded with the AFM were the latest formed (ie. the ”youngest” bond), namely the TCR /pMHC interaction. The experimental procedure gave enough time to other non covalent bonds of the molecular construction used on the cantilever tips to mature, allowing them to reach energetically deeper, hence stronger, bound states [39], [40].

The time available for a TCR to detect a cognate ligand on the APC surface may be crudely estimated as follows : estimating *x* = 10–25 nm to be the maximum distance compatible with molecular interaction, and *D* = 10,000 nm^2^/s to be the diffusion coefficient of pMHC [41], the interaction time between a TCR and a pMHC may be estimated to be approximatively *x^2^/4D* = 0.0025–0.0625 sec. Therefore, the study of very short contact times, such as the ones used here, that do not allow full bond maturation should be highly relevant to the biological problem we investigated.

**Figure 4:**
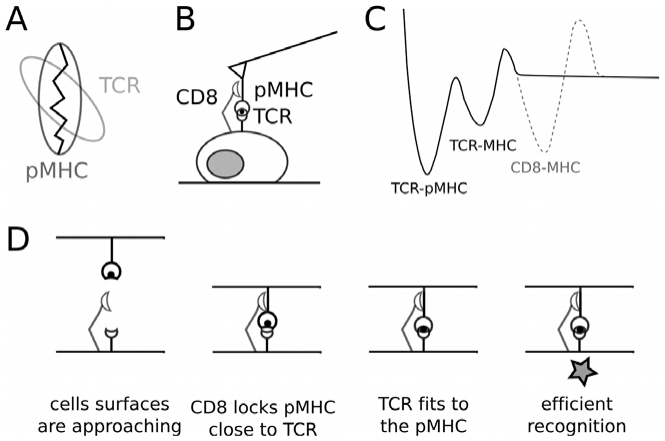
Proposed mechanism. A : Schematics of the optimal configuration of TCR / pMHC recognition, following strutural data. This geometry is complex and may limit the formation of the recognition bridges. B : The cellular case, where the surface molecules are free to move and rotate due to their inclusion in a membrane or to their grafting spacer. This situation is rendered even complex by the presence of surrounding molecules, that can be larger that the TCR as exemplified by the case of CD8. C : The proposed energetical profile of the recognition *E_rec_*, without any force applied to the system, along a suitable reaction coordinate [44]. Several wells may exist, and their access is limited by the exact ”contact time” between the molecules eg. before entering the deeper well of TCR / pMHC close fitting. D. Proposed mechanism of CD8 enhancement of TCR/pMHC recognition. CD8 could help maintain the TCR and pMHC in close proximity to achieve (i) TCR / MHC binding, then (ii) TCR / p fitting, providing a time and distance guidance / locking mechanism, resulting in an addition intermediate well in the energetical pathway (panel C).

We propose that the explanation of the observed results originates from the complex geometry of the recognition bridges that have to form (Fig. 4A) [11], [26], [42], [43]. To recognize a given pMHC, a TCR has to contact both the MHC and the peptide in a very finely controlled way on a restricted set of amino-acids. This implies that the overall optimal geometry of the TCR/ pMHC complex is difficult to achieve, when the molecules are rare and presented in a membrane where they can diffuse and rotate [41], thus decreasing the duration of efficient intermolecular contacts. All together, the molecules by themselves limit the access to the adequate geometry. The existence of a minimal contact time for an efficient TCR/pMHC recognition is consistent with the observed behavior for experimental contact times larger than 0sec. As a consequence, the capacity of our technique to detect the subtle differences between the peptides could be insufficient, leading to the observation of no force difference, but a difference in the adhesion frequency only. In other terms, the effective on-rate of the reaction, between a 2D, membrane bound TCR and a quasi 2D, cantilever bound pMHC could be the limiting parameter [44].

*AF* could be linked to the effective *k_on_* of the recognition following the analysis proposed by [36], while the rupture force is linked to the lifetime of the bond under a given applied force ramp and relates to the off rate, *k_off_*, as dissected experimentally with Dynamic Force Spectroscopy [37], [38]. If these hypotheses were accepted, our results would support the data from [35] where *k_off_* did not strongly vary as a function of the presented peptide, but *A_c_k_on_* varied over three orders of magnitude, *A_c_* being the contact area between a T cell and the model APC used. Morever, this study showed a stronger correlation of activity of the peptide with the on rate than with the off rate. Similarly, other studies [45], [46] found no difference when measuring forces for *multiple* recognition pairs occuring between a T cell and an APC for contact times up to 10 minutes, but reported a difference in adhesive fraction, as a function of the presented peptide. Aside, in a separate set of preliminary experiments, we performed Dynamic Force Spectroscopy on CD8+ cells and we did not observe any variations of the off rate and position of the barrier as a function of the presented peptide (not shown).

Even if the forces were similar in all measured cases, the CD8- case showed that the adhesion frequency did depend on the peptide nature, indicating that our measurements were TCR-specific in that case, which proves their biological significance. The CD8+ case appeared to be more complex, since the system is no more tripartite (TCR/p/MHC) but quadripartite (with CD8 coreceptor) [47]. Because CD8 could be responsible for certain level of adhesion with MHC bearing surfaces in absence of TCR involvment [48], [49], the coreceptor presence could account for two principle points of our results : (i) *AF* did not vary strongly as a function of the presented peptide for CD8+ cells and (ii) *AF* was higher for OVA:H-2K^b^ presented to CD8+ cells in comparison to CD8- ones.

Point (i) might originate from the size difference between the TCR (∼10 nm) and the CD8 (∼15 nm) [50]: this simple geometrical consideration may explain an accessibility difference between both molecules and the pMHC, potentially accounting for the larger *MI* in the pBM1 case. Moreover, it has been observed that the ratio TCR:CD8 (on BM3.3 clones) was on the order of 1:10 (C. Boyer, unpublished results). Such a difference in surface densities of the molecules could decrease the probability of TCR/pMHC recognition events occuring, which may be merged with or masked by the more frequent and geometrically easier CD8/MHC interactions.

We propose to summarize our results in terms of the energy landscape of the TCR/pMHC recognition, as shown on Fig. 4C. Each well represent a degree of recognition, described in Fig. 4D. The energy landscape is complex due to geometrical and environmental reasons. The TCR/pMHC recognition itself could be a double step-in situation, where the first, intermediate well might be due to the necessary close contact between TCR and MHC. Once this state would be optimally achieved, the bond could ”mature” by reaching a deeper well due to the fine fitting of the TCR structure with the presented peptide [39], [51]–[53]. Reaching this next state would be the mechanism allowing T cells to efficiently distinguish between self and non self peptides. The CD8 molecule, which has to interact with the MHC but has a longer extension than the TCR, could assist in reaching this state, by maintaining the TCR within close range of the MHC to which it is itself binding. This would introduce a ”capture and guidance” supplementary well, giving time to the shorter TCR to find the MHC and mature (Fig. 4C,D).

An intriguing possibility would be that the TCR might test, using the cytoskeleton and active cell motion, the pMHC/TCR bond for a force of a few pN (that is below the force resolution of the reported experiments) shortly after formation to increase its sensitivity [17]. Such small forces have been demonstrated to be physiologically relevant [54] and the observed behavior might be correlated to *k_on_* [44].

### Conclusion

Force measurements of TCR / pMHC recognition events at single molecule scale were performed using atomic force microscopy in force mode. When CD8 is absent, the nature of the peptide strongly influences the adhesion frequency. The presence of CD8 strongly modifies this behavior. Importantly, no effect of the peptide on the rupture forces was detected. The proposed explanation originates from the complex geometry and energetical pathway that the molecules have to follow for the peptide to be recognized efficiently, leading to relevant activation outcomes. CD8 could serve as a guidance and "time locking" molecule, to help the close fitting of the TCR and pMHC, by bringing and maintaining them in a sufficiently close range and for a sufficient time to interact. Further investigations, using T cell clones and/or recombinant proteins, could allow one to dissect the relative importance of the fine geometrical constraints (such as the peptide antigen sequence) and of the molecular environment (as exemplified by CD8) in the process of antigenic discremination, the first step in activating the powerful and robust mechanisms of the body protection by the adaptive immune system.

## Materials and Methods

### Commercial reagents

Chemicals for tip and glass surface functionalisation were obtained from Sigma : Bovine serum albumin (BSA), biotinamidocaproyl-labeled (# A6043) ; Streptavidin from Streptomyces avidinii (# S4762) ; Protein G-Biotin from *Streptococcus sp.* (# P8045) ; Poly-L-lysine 0.1% in water (# P8920) ; PBS (as tablets, w/o Ca/Mg). The H-2Kb-Ig recombinant fusion protein (Dimer X, # 550750) was obtained from BD Biosciences. The peptides (OVA : H-SIINFEKL-OH ; pBM1 : H-INFDFNTI-OH) were obtained from Schafer-n. The cell culture medium (RPMI 1640+ L-glutamine) and complements (7% FBS, 1% Hepes 1 M, 1% Pen/Strep, 1% sodium pyruvate, 50 µM β-mercaptoethanol) were obtained from Gibco.

### Cell lines

Alloreactive TCR BM3.3 murine hybridomas were used. TCR BM3.3 recognizes its agonist pBM1/H2-Kb with high avidity in a CD8 co-receptor independent fashion for long term consequences of recognition [27], [28], [43], [55]. The 4C8.98 hybridoma was obtained by fusion between spleen cells from a Rag1-/- BM3.3-TCR-transgenic mice and BW-TCRα-/β-. This hybridoma was selected for expression of the BM3.3 clonotype mAb98 [56], and was further transduced with a genetic construction of the CD8α cDNA chain inserted in the pHbAPr-1 neo vector [57]. The C3.CD8 clone was selected for the 98b clonotype and CD8 surface expressions. It express mainly CD8α and CD8β at cell surface (Fig. S1). These hybridoma are mentionned in the CGG for our group referred 2668. Cell lines were checked for CD3, CD8 and TCR expression and sorted by FACS. Resulting cells were used over one month before been sorted again and were passaged every three days.

### Flow cytometry

Samples containing 10^5^ cells were set in round bottom 96 wells plates with 40 µl of various DimerX dilutions (µg/mL) in FACS Buffer (PBS, FCS 1.5%, EDTA 1 mM, NaN3 0,02%, filtered at 0.22 µm) and incubated 1 h at 4°C under gentle shaking. DimerX were loaded with the desired peptide (OVA or pBM1) following the instructions of the provider (BD Biosciences) at a ratio of 1∶200. After addition of 100 µl FACS Buffer, the cells were centrifugated (1500 rpm, 4 min, 4°C). The 96 wells plate was then flicked and 50 µl biotinylated anti-Mouse Ig (Chemicon International, AP181B) at 1/1000 in FACS Buffer was gently mixed for 30 min at 4°C under gentle shaking. Rinsing was then performed with 100 µl FACS buffer, followed by centrifugation and flicking before addition of 50 µl Streptavidin APC (E-Bioscience, diluted at 1/500 in FACS buffer) for 20 min at 4°C. Following those steps, analysis was immediately performed on living cells. Alternatively, for the analysis of CD3, TCR and CD8, the first step was replaced by incubations of 30 min with similar conditions with anti-CD3biotin (145.2C11, BD Pharmingen), anti-TCRβ (H57, BD Pharmingen or biotinylated anti clonotype 98, made in-house (Buferne 1992) or anti-CD8 pacific blue (E-Bioscience).

### Cell immobilisation

A scalpel-cut PDMS square well of 5 mm×5 mm×1 mm was used to delimitate a zone on plasma activated clean microscope slides [24]. The obtained well was incubated with 100 µL of 0.01% poly-L-lysine for 15 to 30 min. Before the experiment, substrates were gently rinsed with the cell culture medium used to perform the adhesion tests. The PDMS stamp was then removed and a plastic ring (diameter 25 mm, height 10 mm) was glued on the glass slide using vacuum grease. The experiment chamber was then filled with 1 mL of Hepes-buffered culture medium. Diluted cell suspensions were then seeded onto the substrate, let to adhere during 15 to 30 min at room temperature, and gently washed with buffer to remove unbound cells. Using Trypan blue labelling, we observed that the fixation of the cells to the poly-L-lysine was keeping the fraction dead / alive cells to <10%, comparable to what was measured in the cell suspension.

### Atomic force microscope

Cell-tip recognition and mechanical measurements were conducted with an AFM (Nanowizard I, JPK Instruments, Berlin) mounted on an inverted fluorescence microscope (Zeiss Axiovert 200 equipped with 10x and 40x objectives). Bright field imaging was used to select cells and monitor their morphology during force measurements (Fig. S2). The AFM head was equipped with a 15 µm z-range linearized piezoelectric ceramic scanner and an infrared laser. The setup was used in closed height feedback mode [25]. We used Veeco MSCT cantilevers (nominal spring constant k = 10 mN/m, 320 µm long). The sensitivity of the optical lever system was calibrated and the cantilever spring constant were determined in situ using built-in routines of the JPK software before every experiment by using the thermal noise method [58]. The calibration procedure for each cantilever was repeated up to three times to rule out possible errors. Spring constants were found to be consistent with the manufacturer's nominal value (17–22 pN/nm). The AFM and optical microscopes were isolated from ambiant acoustic and mechanical noises using acoustic foam and an active damping table (Halcyonics). All experiments were carried out at 25°C, for no more than an hour, before replacement of the substrate, cell suspension and cantilever.

### Cantilever decoration

We adapted a previously developed protocol (Franz 2007) to the needs of the experiments. Cantilevers were washed in 10% v/v Hellmanex / MQ water at 60°C, then rinced three times in alternating ethanol and water baths before air drying at 60°C, protected from dust. After residual air plasma activation for one minute, they were decorated sequentially using biotin-BSA (0.5 mg/mL in NaHCO3 100 mM, pH 8,6 ; overnight), streptavidin (0.5 mg/mL in PBS w/o Ca^2+^/Mg^2+^, pH 7.4 ; 45 min), biotin-protein G (0.5 mg/mL in PBS w/o Ca^2+^/Mg^2+^, pH 7.4 ; 45 min) and finally Dimer X (0.01 mg/mL in PBS w/o Ca^2+^/Mg^2+^, pH 7.4 ; 3 h). Between each step, the levers were washed intensively three times in PBS to remove unbound proteins. The functionalized levers were then incubated in an excess of peptide following the instructions of the provider (at least 200 to 2000 times more peptide than Dimer X, at 4°C, in PBS, overnight). The levers were kept up to three days in this solution until final rincing prior to use. This process ensures that all intermediate bonds can consolidate sufficiently for the measured rupture forces to be mainly attributed to the pMHC end of the molecular sandwich [39]. To qualitatively assess that the molecular construction built up on the lever was present, several tests using either fluorescent proteins or antibody labelling were performed. Using a fluorescein labelled streptavidin, the fluorescence level was very weak without the biotin-BSA compared to the case where this preliminary layer was present. The presence of H-2K^b^ dimers was checked by using an FITC labelled anti-MHC antibody (20.8.4, gift from A. Guimezanes, CIML, Marseille). Compared to the case without MHC, the fluorescence level of the case with MHC was 3-fold higher, indicating the good functionalization of the levers with the desired ”final” molecules (not shown). One has to note that it is technically difficult to dilute and measure precisely the density of molecules on the AFM sharp tip, hence no precise quantification of the number of molecules is provided here.

### Adhesion measurements

Using the optical microscope, a calibrated cantilever is positioned over a chosen cell (Fig. S2). The speed for bringing to or removing the tip from cell surface was set to *v* = 1 µm/sec and the desired contact force to 50 pN. Contact force cannot be decreased to lower levels to minimize both the contact time and area without compromising a frequent and reproducible tip to cell contact. At least 2048 deflection data points were collected over a pulling distance of 500 nm to obtain a force curve. These parameters ensured that more than 90% of the acquired force curves will show a clear contact between the tip and the cell, and that this contact will be the gentlest possible. The time resolution (∼1 msec) is sufficient to record the molecular unbinding events. The contact time was set to 0 sec before tip retraction, leading to an effective contact time between the tip and the cell surface, because of the deformability of this latter, on the order of 100 to 250 msec. For each condition, at least 17 cells and 154 force curves were examined over several days of culture (Table 1).

#### Data processing

Each detachment curve was examined by eye and processing was performed using to the built-in JPK-IP software using force curve batch processing procedures : correcting for baseline shift and/or tilt, then applying a sliding average box of 15 to 45 points to detect the force jumps when present. Force jump magnitudes were recorded and pooled to calculate mean and SD. ANOVA + Tukey post test was used using Prism (GraphPad Software). In addition to the number of measured force curves, *N_curves_* , the number of the curves presenting at least one identified unbinding event, *N_adhesion_*, together with the number of unbinding events each presents, *N_jumps_*, were recorded. The adhesion frequency, *AF = N_adhesion_/N_curves_* for each cell was calculated, and then the average and SEM over the different cells were obtained. t-tests were used to examine the significance of the observed *AF* differences using Prism (GraphPad Software). Additionaly, we calculated an index of multiplicity of the unbinding events, *MI = N_jumps_/N_adhesion_* . This index reveals the fraction of multiple adhesion events recorded for each condition.

## Supporting Information

Figure S1 **Flow cytometry experiments.** Biotynylated H35.17.2 mAb specific for CD8β [59], and 53.6 specific for CD8α (BD Pharmingen) were used to characterize the CD8 constituants of the C3.CD8 cell surface. 75% of C3.CD8 hybridoma express the dimer CD8αβ. Comparison with naive CD8 T cell from mouse lymph nodes suggest that at the cell surface of C3.CD8 the α chain is two times more abundant than at the surface of naive CD8 T cells. This suggests that the dimer CD8αβ coexists with CD8αα at the C3.CD8 cell surface.
(TIF)

Figure S2 **Micrographs from the experiments.** Decorated lever positionned over a dispersed population of T hybridomas, attached to the polylysine coated coverslide. Insert : the pyramidal tip, at the bottom end of the lever, is positionned over a healthy cell. Bar = 20 µm.
(TIF)

Figure S3 **AFM micromechanical experiments.** A : Schematics of the mechanical measurements by indentation of polylysine adhered T hybridomas using an unfunctionnalized AFM lever The tip is used to indent the cell until a prescribed contact force is reached (50 or 500 pN). B : Typical indentation force curve (*F* vs. tip sample separation ie. indentation [21], [31] – pushing, black and pulling, grey) for a contact force of 50 pN, a contact time of 0 sec and at a speed of *v_press_ = v_pull_* = 1 µm/sec. Such a force curve was used to measure the Young modulus, *E*, of the cells using a fit based on the Hertz model for a pyramidal indenter (white line). C : Young modulus, *E*, as a function of cell type and contact force. At least 10 cells, and more than 125 force curves per condition were used to determine the mean and SD for *E*.
(TIF)

Figure S4 **AFM force mode experiments.** A. Example of a force curve showing two separate unbinding events. The white line is a 45 pts average used to automatically detect and measure the jumps position and magnitude (vertical grey lines). B. Comparison of the mean force (+/− SD) of the two sucessive jumps for the case pBM1 vs. C3.CD8. No significant difference was observed as assessed by a Mann-Whitney test. C. Plot of magnitudes of the first jump vs. the second. No tendancy is apparent. D. Plot of the magnitude of the force jumps vs. the distance between them. E. Distribution of distance between the first and the second jump. The mean is 86.4 nm and the SD is 68.9 nm. F. Subset of data from panel B. Average forces for successive jumps having a distance <25 nm, ie. similar to full separation of TCR/CD8/pMHC. No significant difference was observed as assessed by a Mann-Whitney test.
(TIF)

Figure S5 **AFM force mode experiments.** A–C : Adhesion frequency, *AF* (+/− SEM), per cell when varying the contact time, keeping the contact force at 50 pN. A : pBM1 peptide ; B : OVA peptide ; C : no peptide. Closed (open) symbols are for CD8+ (CD8-) cells. In the case of BW cells, lacking TCR and CD8 molecules, *AF* was found lower in all examined conditions (i) pBM1 : 14.4+/−1.9% for 0 sec, 29.8+/− 10.5 % for 100 msec ; 28.0+/−13.6% for 1 sec ; (ii) OVA : 10.6+/− 1.9% for 0 sec ; (iii) no peptide : 17.1+/−4.8% for 0 sec, 21.0+/−3.7% for 100 msec, 20.8+/−4.7% for 1 sec. D : Average rupture force of single complex ruptures, extracted from the histograms (+/− SD), as a function of cell type and peptide. The values are *not* significantly different (ANOVA + post-test, *p*>0.05). 5–10 cells, resulting in 42–90 force curves per condition, were examined. The data for 0 sec contact time is the same as the one presented on Fig. 3.
(TIF)
