# Supplementary figures and images for: Force Measurements of TCR/pMHC Recognition at T Cell Surface
Source: PLoS One. 2011 Jul 22;6(7):e22344. doi: 10.1371/journal.pone.0022344 (PMC3142151; doi:10.1371/journal.pone.0022344)

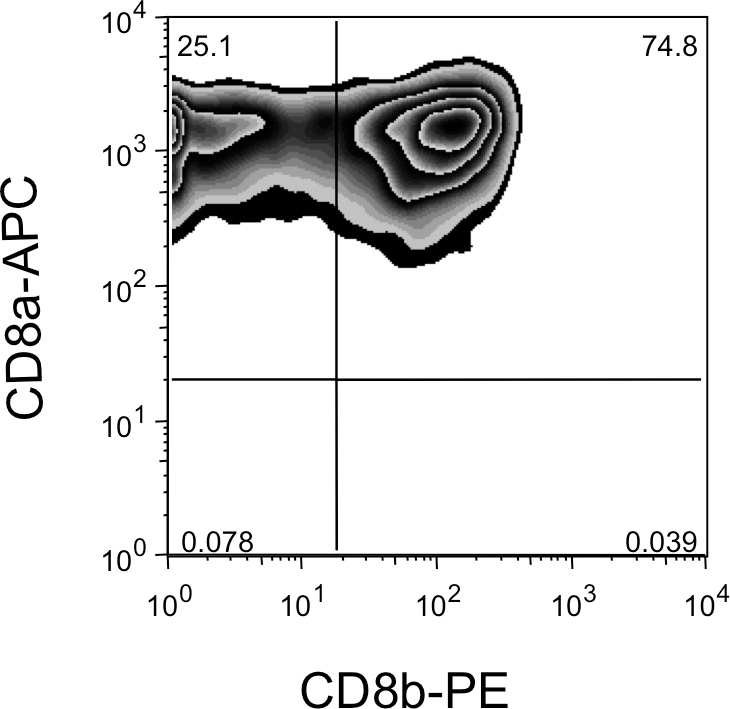

Supplement: Figure S1 — Flow cytometry experiments. Biotynylated H35.17.2 mAb specific for CD8β [59], and 53.6 specific for CD8α (BD Pharmingen) were used to characterize the CD8 constituants of the C3.CD8 cell surface. 75% of C3.CD8 hybridoma express the dimer CD8αβ. Comparison with naive CD8 T cell from mouse lymph nodes suggest that at the cell surface of C3.CD8 the α chain is two times more abundant than at the surface of naive CD8 T cells. This suggests that the dimer CD8αβ coexists with CD8αα at the C3.CD8 cell surface. (TIF) [file pone.0022344.s001.tif]

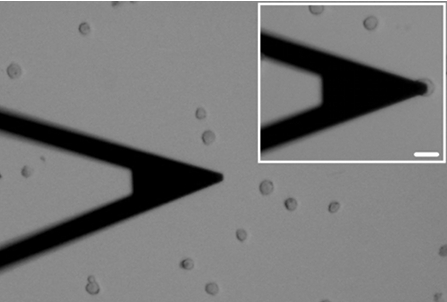

Supplement: Figure S2 — Micrographs from the experiments. Decorated lever positionned over a dispersed population of T hybridomas, attached to the polylysine coated coverslide. Insert : the pyramidal tip, at the bottom end of the lever, is positionned over a healthy cell. Bar = 20 µm. (TIF) [file pone.0022344.s002.tif]

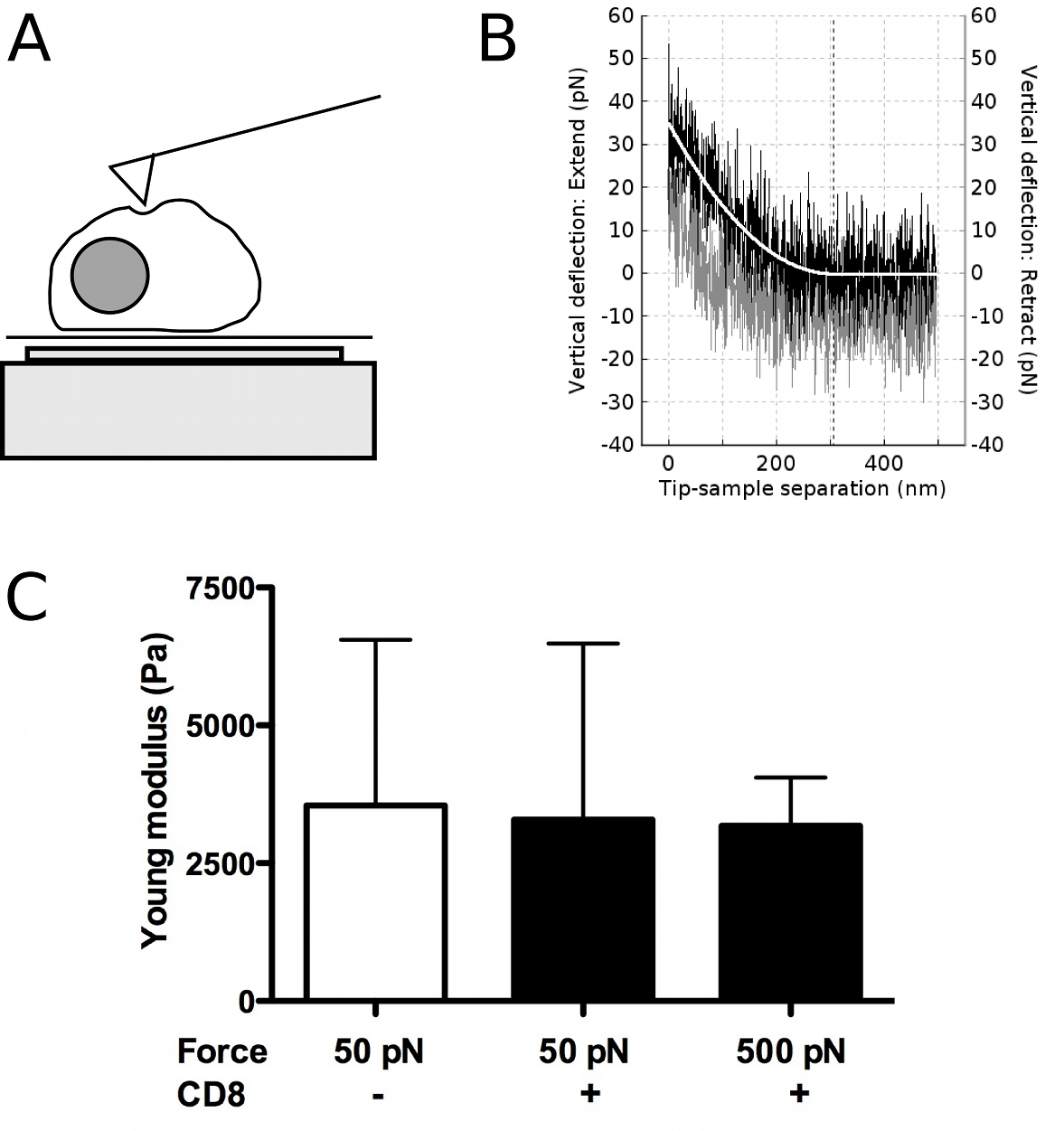

Supplement: Figure S3 — AFM micromechanical experiments. A : Schematics of the mechanical measurements by indentation of polylysine adhered T hybridomas using an unfunctionnalized AFM lever The tip is used to indent the cell until a prescribed contact force is reached (50 or 500 pN). B : Typical indentation force curve (F vs. tip sample separation ie. indentation [21], [31] – pushing, black and pulling, grey) for a contact force of 50 pN, a contact time of 0 sec and at a speed of vpress = vpull = 1 µm/sec. Such a force curve was used to measure the Young modulus, E, of the cells using a fit based on the Hertz model for a pyramidal indenter (white line). C : Young modulus, E, as a function of cell type and contact force. At least 10 cells, and more than 125 force curves per condition were used to determine the mean and SD for E. (TIF) [file pone.0022344.s003.tif]

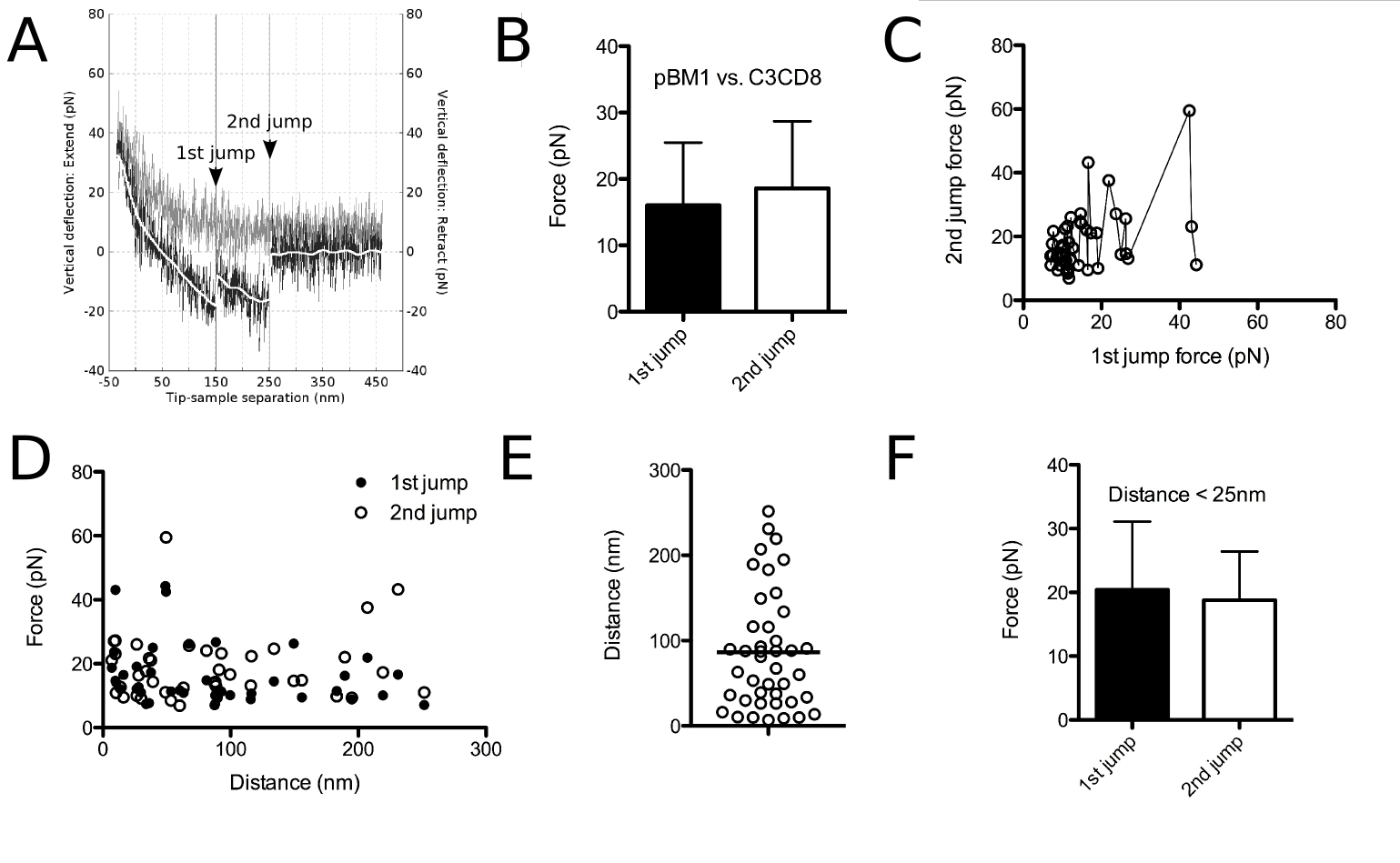

Supplement: Figure S4 — AFM force mode experiments. A. Example of a force curve showing two separate unbinding events. The white line is a 45 pts average used to automatically detect and measure the jumps position and magnitude (vertical grey lines). B. Comparison of the mean force (+/− SD) of the two sucessive jumps for the case pBM1 vs. C3.CD8. No significant difference was observed as assessed by a Mann-Whitney test. C. Plot of magnitudes of the first jump vs. the second. No tendancy is apparent. D. Plot of the magnitude of the force jumps vs. the distance between them. E. Distribution of distance between the first and the second jump. The mean is 86.4 nm and the SD is 68.9 nm. F. Subset of data from panel B. Average forces for successive jumps having a distance <25 nm, ie. similar to full separation of TCR/CD8/pMHC. No significant difference was observed as assessed by a Mann-Whitney test. (TIF) [file pone.0022344.s004.tif]

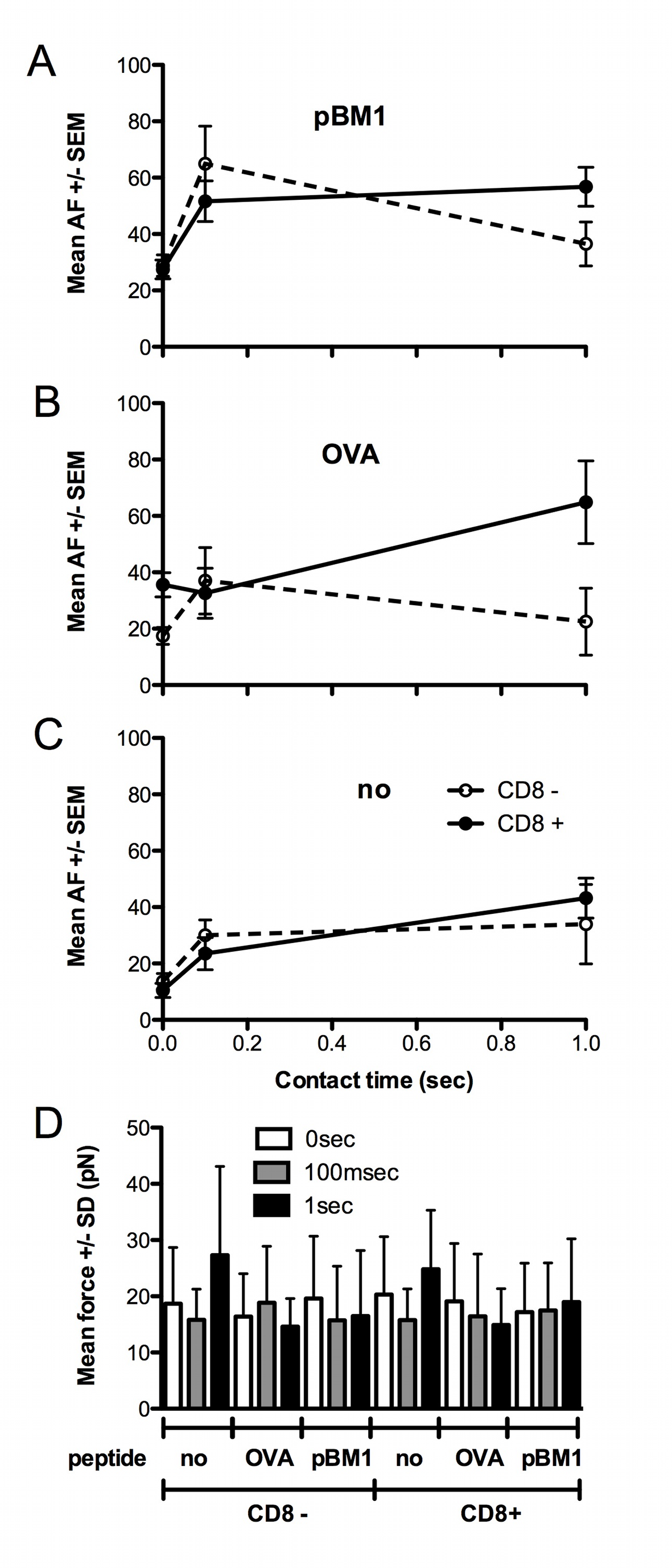

Supplement: Figure S5 — AFM force mode experiments. A–C : Adhesion frequency, AF (+/− SEM), per cell when varying the contact time, keeping the contact force at 50 pN. A : pBM1 peptide ; B : OVA peptide ; C : no peptide. Closed (open) symbols are for CD8+ (CD8-) cells. In the case of BW cells, lacking TCR and CD8 molecules, AF was found lower in all examined conditions (i) pBM1 : 14.4+/−1.9% for 0 sec, 29.8+/− 10.5 % for 100 msec ; 28.0+/−13.6% for 1 sec ; (ii) OVA : 10.6+/− 1.9% for 0 sec ; (iii) no peptide : 17.1+/−4.8% for 0 sec, 21.0+/−3.7% for 100 msec, 20.8+/−4.7% for 1 sec. D : Average rupture force of single complex ruptures, extracted from the histograms (+/− SD), as a function of cell type and peptide. The values are not significantly different (ANOVA + post-test, p>0.05). 5–10 cells, resulting in 42–90 force curves per condition, were examined. The data for 0 sec contact time is the same as the one presented on Fig. 3. (TIF) [file pone.0022344.s005.tif]
